# Slanted recession on bilateral lateral rectus for the treatment of intermittent Exotropia with convergence insufficiency

**DOI:** 10.1186/s12886-022-02367-1

**Published:** 2022-03-24

**Authors:** Yueping Li, Huiyu Lin

**Affiliations:** 1 Tianjin Eye Hospital, Clinical College of Ophthalmology of Tianjin Medical University, Tianjin Key Laboratory of Ophthalmology and Vision Science, Tianjin, 300020 China; 2 QuanZhou Women’s and Children’s Hospital, Quanzhou, 362000 China

**Keywords:** Intermittent exotropia (XT), Convergence insufficiency (CI), Slanted recession, Near-distance difference (NDD)

## Abstract

**Purpose:**

To evaluate the efficacy and safety of slanted bilateral lateral rectus recession (S-BLRc) for the treatment of convergence insufficiency-type intermittent exotropia (CI-IXT) in children and to probe the relationship of the slanted amount and surgical outcomes.

**Methods:**

Retrospective study. Fifty-eight patients with CI-IXT, aged 4 to 10 years old, underwent S-BLRc procedures. According to the different slanted amount between the upper and lower poles of lateral rectus, all the patients were grouped: Group A (slanting 1 mm, *n* = 22), Group B (slanting 1.5 mm, *n* = 18) and Group C (slanting 2 mm, *n* = 18). The successful surgical outcome was defined as deviation in the primary position ranging from exotropia< 8^△^ to esotropia< 5^△^ both at near and at distant as well as the near-distance difference (NDD) < 5^△^. We analyzed and compared the preoperative and postoperative data including deviations both at near and at distance, NDD, objective torsion, horizontal deviation at up and down gaze, lateral incomitance, binocular vision and surgical success rate among three groups.

**Results:**

The average deviations were significantly decreased from − 37.1^△^ ± 4.2^△^ (−,exotropia) to − 1.4^△^ ± 4.6^△^ at near (*P* < 0.05) and from − 25.8^△^ ± 3.7^△^ to − 0.1 ± 4.1^△^ at distance (*P* < 0.05). The postoperative NDD on average was significantly reduced from 10.0^△^ to 1.8^△^ in Group A (*P* < 0.05), from 11.2^△^ to 0.8^△^ in Group B (*P* < 0.05) and from 13.3^△^ to 0.9^△^ in Group C (*P* < 0.05). There was a significant difference in the mean corrections of NDD among the three groups (8.2^△^ in group A, 10.3^△^ in group B and 12.4^△^ in group C respectively, *P* < 0,05). All the patients attained various improvement of stereopsis after surgery. None had torsional diplopia, A-V pattern and lateral incomitance after strabismic surgery. Totally, the surgical success rate was 89.7% in our series at the 6- to 8-month follow-up.

**Conclusions:**

Slanted bilateral lateral rectus recession is an effective and safe procedure for the treatment of CI-IXT in children. S-BLRc can successfully collapse exotropia both at distance and at near, decrease NDD and benefit to gain binocular vision. The correction of NDD was associated with the slanted amount.

## Introduction

Convergence insufficiency intermittent exotropia (CI-IXT) is a subtype of intermittent exotropia characterized by a greater deviation of at least 10^△^ at near than that at distance [1–3]. Reducing the near-distance difference (NDD) is imperative to effective treatment of CI-IXT. A medial rectus (MR) strengthening procedure, which can improve convergence and reduce the NDD, has been recommended for treatment of CI-IXT [4–8]. However, it has not only been associated with persistence postoperative overcorrection at distance for a few months, but also with unsatisfactory undercorrection in the long-term [5]. Kushner found an undercorrection rate of 83% 1 year after symmetrical MR resections [9]. With regard to MR resection and lateral rectus (LR) recession (R&R) procedure for the treatment of CI-IXT, researchers have suggested that the resection amount was determined by the deviation at near and the recession amount was based on the deviation at distance [8, 10]. Although NDD collapsed around 10^△^, 7 to 18% of patients could be overcorrected, at a success rate of 42% 1 year after surgery [10, 11]. With regards to LR recession procedure for CI-IXT, previous studies have shown that the surgical amount depending on the deviation at distance is prone to undercorrection but the surgical amount according to the deviation at near could cause over-correction [12, 13] . However, standard LR recession does not effectively reduce the NDD [14, 15]. Some researchers suggested that slanted recession of the LR is an effective surgical procedure for reducing deviation at distance and at near as well as collapsing the NDD [14–18]. To date, however, the effect of different slanted amounts on postoperative outcomes remains unclear. In this study, we aimed to investigate the relationship between slanting recession amount and surgical outcomes and evaluated efficacy and safety of different amounts of slanting recession for the treatment of CI-IXT in children.

## Subjects and methods

### Patients

All the surgical procedures were performed in accordance with the Declaration of Helsinki for research involving human subjects. The study protocol was approved by the Institutional Review Board (IRB) of Tianjin Eye Hospital. All the participants signed written informed consent. Informed consent for minors was obtained from their legal guardian(s). A total of 58 children, aged between 4 to10 years old, with CI-IXT were enrolled from August 2019 to July 2020. Patients who exhibited high AC/A (gradient method), A-V pattern of strabismus, vertical deviation≥5^△^, torsional strabismus, amblyopia, nystagmus, previous strabismic surgery were excluded.

### Examinations

All patients underwent complete ophthalmologic evaluations, including best corrected visual acuity, cycloplegic refraction, fundus examination, ocular movement, AC/A ratio and the deviations measured with prism and alternating cover test (PACT), before and after operations. Maximum deviations at near and at distance were determined after patching one eye for 60 min. Stereopsis was evaluated by the Titmus test. The objective torsion was measured on the fundus photograph using CorelDrawl X7 software (Corel Corporation, Canada). The fovea-disc angle was defined as positive (+) if the macula located above the horizontal line passing through the center of the optic disc and negative (−) when macula located below the horizontal line. We took the sum of the bilateral fovea-disa angles as the total objective torsional angle so as to avoid the effect of the head tilting on the ocular torsion. The final follow-up visits were conducted 6 to 8 months after surgeries.

### Surgical procedure and grouping

**Fig. 1:**
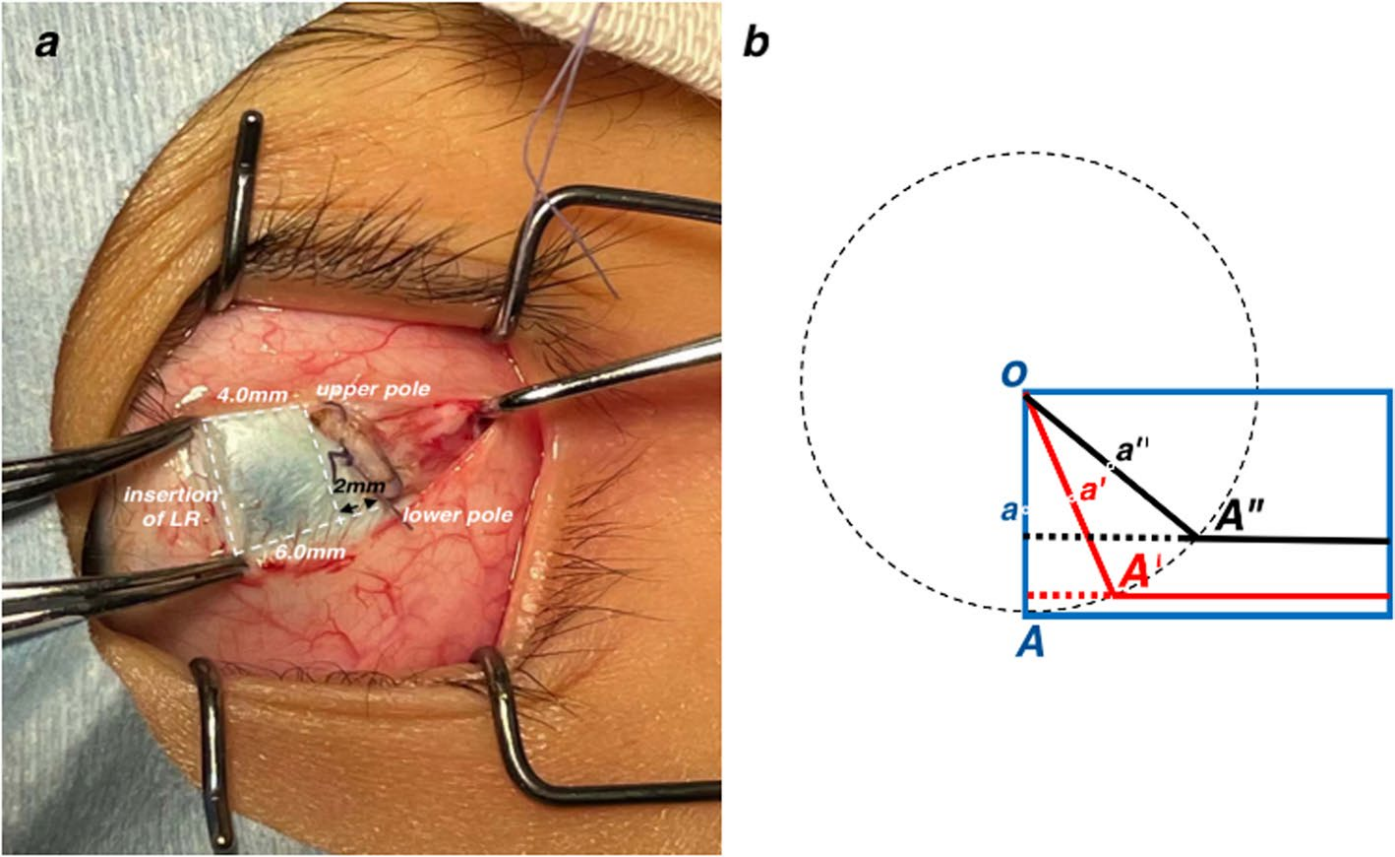
The procedure of slanted recession of LR. **a** Taken during the surgery, recession 4 mm of the upper pole and recession 6 mm of the lower pole. The slanted amount is 2 mm. **b** The diagram of slanted recession procedure. O is the opper pole of LR. OA (blue) is the new insertion and a (blue) is the central point of OA in the standard recession procedure. During the procedure in our study, the scleral pass of the upper pole of LR was firstly performed and then the scleral pass of the lower pole. OA’ (red) is the slanted insertion and a’ (red) is the central point of OA’ in the 1 mm slanted recession procedure. OA” (black) is the slanted insertion and a” (black) is the central point of OA” in the 2 mm slanted recession procedure. To keep the original length of LR insertion, the actual width of LR, perpendicular to the direction of the muscle force, is narrowed and the central point of new insertion is shifted upwards a bit

All surgical procedures were performed by one surgeon. The procedure for slanted recession of LR is illustrated in Fig. 1a. Summarily, the upper poles of LR were recessed 4 to 6 mm according to the deviations at distance while the lower poles were recessed 6 to 8 mm according to the deviation at 33 cm. The slanted amount was defined as the recession difference between the lower pole and upper pole. All patients were grouped based on the slanted amount as follows: Group A-1 mm; Group B-1.5 mm and Group C-2 mm. In this study, the criteria for successful surgical outcome comprised postoperative deviation ranging from exotropia < 8^△^ to esotropia < 5^△^ both at distance and at near as well as NDD < 5^△^.

### Statistical analysis

The preoperative and postoperative data were compared using paired t-test. The data among the groups were compared using one-way ANOVA. The improvement of stereopsis and surgical success rate among the groups were analyzed using Fisher’s Exact Test. All data were analyzed with IBM SPSS Statistics 23.0 (IBM, Armonk, NY, USA). *P* values less than 0.05 were deemed statistically significant.

## Results

**Fig. 2:**
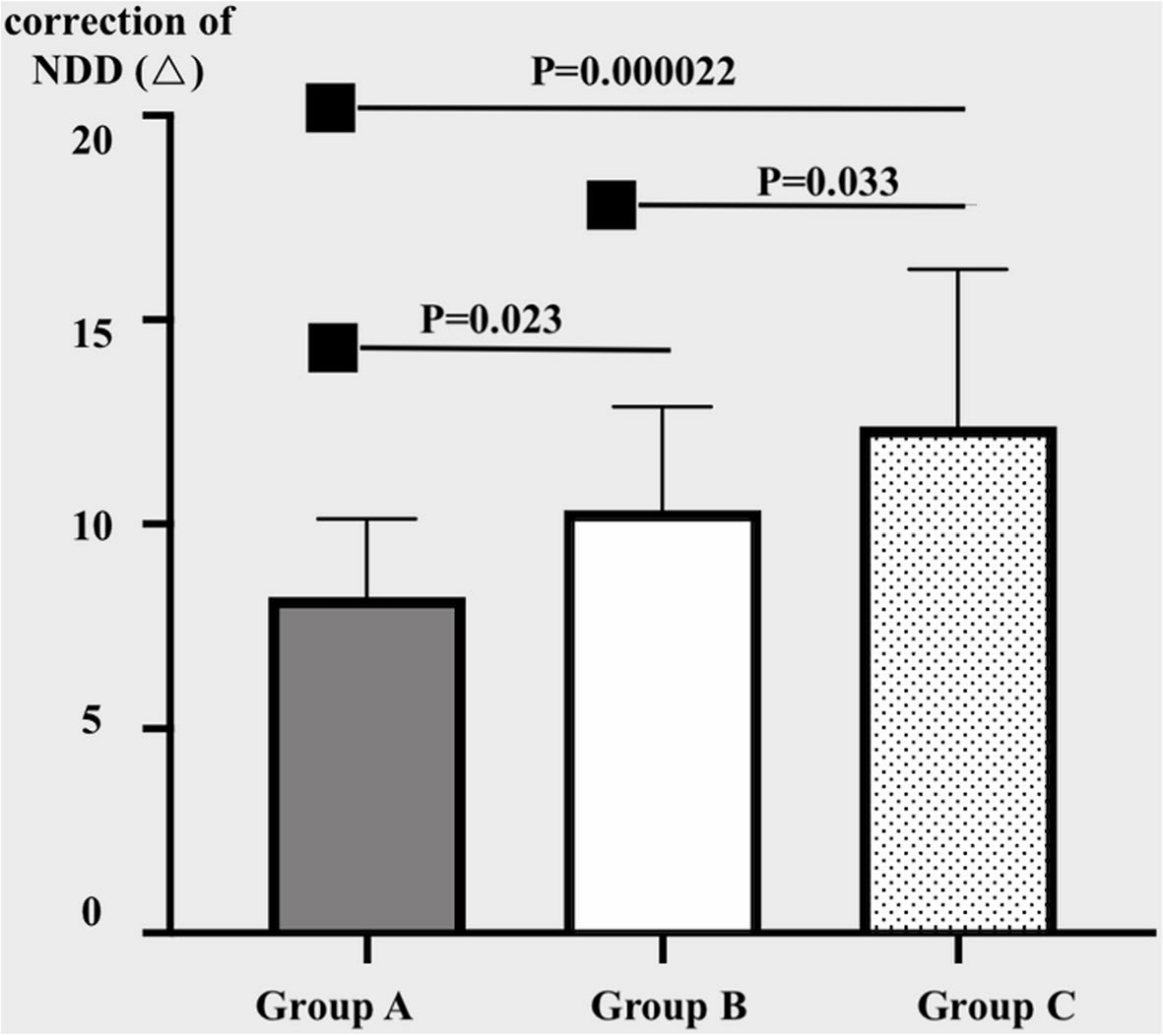
The corrections of NDD in the three groups

Fifty-eight patients, 33 males and 25 females, aged between 4 to 10 years old (average 6.53 ± 2.35 years) were enrolled in this study. The average deviations significantly decreased from − 37.1^△^ ± 4.2^△^at near preoperatively to − 1.4^△^ ± 4.6^△^ postoperatively (*P* < 0.05) and from − 25.8^△^ ± 3.7^△^ at distance preoperatively to − 0.1^△^ ± 4.1^△^ postoperatively (*P* < 0.05). The mean NDD also significantly reduced from 11.4^△^ ± 2.0^△^ to 1.2^△^ ± 2.2^△^ (*P* < 0.05). Deviations at both near and distance as well as NDD exhibited a significant decrease after surgery in each group (Table 1). We also found statistically significant differences in the postoperative deviations at near and corrections of NDD among the three groups (*P* = 0.039, 0.00011). By pairwise comparison, NDD was corrected most in Group C and least in Group A (Fig. 2). The correction of NDD (Y) was significantly correlated with the slanted amount (X) (R = 0.53, *P* = 0.000018), with the following fitting equation: Y = 4.163X + 4.071.

**Table 1:** Preoperative and postoperative deviation at near and at distance and NDD in the three groups

	Deviation at Near (^△^)			Deviation at Distance (^△^)			NDD(^△^)			Correction of NDD (^△^)	Success rate
	Preop	Postop	*P*	Preop	Postop	*P*	Preop	Postop	*P*		
Group A (n = 22)	−36.5 ± 2.8 (− 34 ~ − 40)	−3.2 ± 5.4 (− 18 ~ + 4)	6.28E-18	− 26.7 ± 2.4 (− 25 ~ − 30)	−1.6 ± 4.3 (− 14 ~ + 4)	9.10E-17	10.0 ± 0.0 (10 ~ 10)	1.8 ± 1.9 (0 ~ + 6)	2.78E-15	8.2 ± 1.9 (4 ~ 10)	90.9% (20/22)
Group B (*n* = 18)	−36.8 ± 4.6 (− 30 ~ − 45)	− 0.9 ± 3.4 (− 10 ~ + 6)	1.31E-15	−25.7 ± 3.9 (− 20 ~ − 32)	− 0.1 ± 2.4 (− 4 ~ + 5)	3.58E-15	11.2 ± 1.5 (10 ~ 15)	0.8 ± 2.1 (− 4 ~ + 6)	3.35E-12	10.3 ± 2.5 (4 ~ 14)	88.9% (16 /18)
Group C (*n* = 18)	− 38.2 ± 5.2 (− 30 ~ − 50)	+ 0.4 ± 3.7 (− 4 ~ 14)	1.54E-18	−24.8 ± 4.5 (− 20 ~ − 35)	+ 1.6 ± 4.6 (− 4 ~ + 16)	1.77E-10	13.3 ± 2.2 (10 ~ 15)	0.9 ± 2.5 (−4 ~ + 8)	1.38E-10	12.4 ± 3.9 (2 ~ 17)	88.9% (16/18)
*P*	0.415	0.039		0.285	0.490		1.52E-8	0.321		0.00011	0.452

Deviation: -, exotropia; +, esotropia

Notably, we found no statistically significant differences between preoperative and postoperative variables, including total fovea-disa angle, difference of deviation at up and down gaze, lateral incomitance in each group. A similar trend was observed among groups (Table 2).

**Table 2:** The preoperative and postoperative results of the total fovea-disa angle, Up-down gaze deviation, Right side, Left side before and after surgery

	Total fovea-disa angle (°)			difference of deviation between up and down gaze ^a^ (^△^)			lateral incomitance at right gaze ^b^ (^△^)			lateral incomitance at left gaze ^b^ (^△^)		
	Preop	Postop	*P*	Preop	Postop	*P*	Preop	Postop	*P*	Preop	Postop	*P*
Group A (*n* = 22)	−11.4 ± 4.95	− 10.8 ± 4.48	0.519	− 1.23 ± 2.45	−0.82 ± 1.92	0.488	− 0.32 ± 1.99	− 0.68 ± 1.84	0.589	− 0.55 ± 1.60	− 0.73 ± 2.03	0.758
Group B (n = 18)	−11.8 ± 4.97	− 13.2 ± 3.43	0.264	− 1.53 ± 3.52	− 1.13 ± 1.89	0.710	0.27 ± 2.76	− 0.73 ± 1.53	0.231	0.13 ± 1.73	−0.47 ± 1.96	0.384
Group C (n = 18)	−10.5 ± 6.55	−12.6 ± 3.41	0.213	−1.55 ± 4.50	0.18 ± 3.52	0.430	−0.64 ± 3.53	−0.18 ± 0.75	0.676	−0.27 ± 3.41	− 0.73 ± 1.68	0.708
*P*	0.911	0.370		0.950	0.359		0.669	0.623		0.648	0.911	

^a^the deviation at up gaze minus the deviation at down gaze

For example, 8^△^ exotropia(−8^△^) at up gaze and 6^△^ (−6^△^) exotropia at down gaze, a = (− 8^△^) -(− 6^△^) = −2^△^

6^△^ exotropia (− 6^△^) at up gaze and 8^△^ (− 8^△^) exotropia at down gaze, a = (− 6^△^) -(− 8^△^) = 2^△^

^b^the deviation in the primary position minus the deviation at the right or the left gaze

For example, 8^△^ exotropia(− 8^△^) in the primary position and 6^△^ (− 6^△^) exotropia at the left gaze, b = (− 8^△^) -(− 6^△^) = − 2^△^

At 6- to 8-month follow-up visit, the success rate was 89.7% (52/58) in our series. Notably 3 patients (5.2%) had over-corrected and 3 patients (5.2%) had under-correction. There were no significant difference in surgical success rate among the three groups (Z = 3.72, *P* = 0.452) (Table 1). However, the constituent ratio of the number of patients with different stereopsis was significantly different before and after surgery (Z = 18.82, *P* = 0.001) (Table 3.). Notably, the number of patients with fine stereopsis (≤60″) increased from 11 to 35 after surgery. Moreover, 3 patients with undercorrection after surgery also exhibited improved binocular vision. However, the 3 patients with overcorrection exhibited no stereopsis due to diplopia.

**Table 3:** The number of patients with different stereopsis before and after surgery

	Titmus≤60”	60″<Titmus≤200”	200″<Titmus≤400”	400″<Titmus≤3000”	No stereopsis
Preoperation (*n* = 58)	11	24	12	5	6
Postopration (*n* = 58)	35	14	6	3	3

Three cases without stereopsis were overcorrected after surgery

## Discussion

Horizontal strabismus subtypes that may be attributable to abnormal compartmental function include near/distance disparity esotropia or exotropia, distance esotropia or exotropia, and A- or V-pattern strabismus [15, 19, 20]. Both slanted recession and slanted resection of the horizontal rectus were first described for treatment of A-V pattern strabismus [19]. Scott demonstrated that the upper and lower margins of lateral rectus (LR) have the same length in the primary position, although the lower margin is shorter than the upper margin when gazing down at near [21]. Previous studies have shown that the lower part of LR fibers can affect XT deviation at both near and downgaze. Particularly, the LR muscle shows the highest level of compartmental organization [20]. Demer found that in the LR muscle, the abducens nerve divides into both superior and inferior portions, which do not overlap and generate the asymmetric contraction on the eye-globe [22]. Conversely, other reports have shown that both superior and inferior compartments of the lateral rectus muscle relax symmetrically in adduction and convergence [23]. Different operations can be designed for the upper and lower parts of the muscle lateral rectus, although the underlying mechanism remains unknown. Snir et al. first introduced slanted recession of LR recession for CI-IXT. They recommended that the surgical amount of the lower and upper poles were dependent on exodeviation at near and at distance, respectively [15]. Notably, NDD reduced from 14^△^ to 2.9^△^ by balancing of the upper and lower muscle tension [14], the surgical successful rate varied from 58 to 92% whereas the NDD correction per millimeter of slanted amount varied from 4.6^△^ to 8.7^△^ in the previous literatures [15–18]..

In our series, we found a surgical success rate of 89.7% at 6 to 8 months of follow-up. Notably, 2 and 1 patient from group A and B, respectively, were undercorrected, whereas 2 and 1 from groups C and B, respectively, were overcorrected. Based on these outcomes, we hypothesize that a smaller and larger slanting recession may have given rise to more undercorrections and more overcorrections, respectively.

Our results also confirmed that S-BLRc could successfully reduce NDD. Particularly, we recorded a mean NDD correction of 10.2^△^, which was similar to that reported in previous studies [14–18]. The NDD corrections were 8.2 ± 1.9^△^, 10.3 ± 2.5^△^ and 12.4 ± 3.9^△^ in groups A, B and C, respectively, and these were significantly different. Although there were only three groups in our study, we generated an equation of NDD correction (Y) and slanted amount (X); Y = 4.163X + 4.07. Based on this equation, it was evident that the NDD correction per 1 mm slanting in our series was lower than that observed in other studies. We speculated that the procedure of S-BLRc in our study not only caused the change in the more posterior insertion of the inferior pole of LR, but also narrowed the width of LR and shifted LR upward a bit, compared to the procedure of standard recession (Fig. 1b). All factors had different impacts at near and at distance. The rang of the slanted amount, from 1 to 2 mm in our series, was small, thus further studies on larger slanted recession based on more groups are needed to validate these results and obtain a more accurate equation of the relationship between the NDD correction and slanted amount.

Results from a study on rabbit eye with slanted recession of superior rectus revealed that both margins had anterior movement at 3 months post-operation, indicative of a decrease in surgical effectiveness [24]. Notably, the decrease in slanting degrees was more in the 4 mm than that in the 2 mm group. However,the collapsing of NDD after S-BLRc maintained well over postoperative 6 months in our study. Even the 6 patients with both undercorrection and with overcorrection, still had smaller postoperative NDDs relative to those at preoperation. Based on these findings, we suggest that the effect of S-BLRc on NDD improvement may be relatively stable.

Previous studies have shown that 13.6% of patients who underwent S-BLRc exhibited secondary esotropia at downgaze due to postoperative V-pattern, while 4.5% of them developed postoperative A pattern [13]. In our series, no patient developed the A-V phenomenon after surgery. We attribute this phenomenon to small slanting in our study, as well as lack of A or V pattern before surgery. We also measured the objective torsion on the fundus photograph, but not the subjective torsion. No patient developed postoperatively torsional diplopia in our series, even in those with either overcorrection or undercorrection. Moreover, no patient developed postoperative lateral incomitance in the present study.

This study had some limitations. Firstly, NDDs were relatively small, which might explain the slanting amount ranged from 1 to 2 mm. Secondly, the patients were followed up only for a period of 6 to 8 months after surgeries. Future studies, using larger sample sizes and larger slanted recession as well as longer follow up times are needed to validate these findings.

In conclusion, S-BLRc is an effective and safe procedure for the treatment of CI-IXT in children, owing to its efficacy in correcting deviation both at distance and at near. S-BLRc can effectively reduce NDD and improve binocular visual function.

## Data Availability

Data used in the study is provided under supplementary material section.
